# ACE2-like enzyme B38-CAP suppresses abdominal sepsis and severe acute lung injury

**DOI:** 10.1371/journal.pone.0270920

**Published:** 2022-07-22

**Authors:** Takafumi Minato, Tomokazu Yamaguchi, Midori Hoshizaki, Satoru Nirasawa, Jianbo An, Saori Takahashi, Josef M. Penninger, Yumiko Imai, Keiji Kuba

**Affiliations:** 1 Department of Biochemistry and Metabolic Science, Akita University Graduate School of Medicine, Akita, Japan; 2 Laboratory of Regulation of Intractable Infectious Diseases, National Institute of Biomedical Innovation, Health and Nutrition (NIBIOHN), Ibaraki, Osaka, Japan; 3 Biological Resources and Post-Harvest Division, Japan International Research Center for Agricultural Sciences, Tsukuba, Ibaraki, Japan; 4 Akita Research Institute of Food and Brewing, Akita, Japan; 5 Department of Medical Genetics, Life Science Institute, University of British Columbia, Vancouver, BC, Canada; Hualien Tzu Chi Hospital, TAIWAN

## Abstract

Angiotensin-converting enzyme 2 (ACE2) is the carboxypeptidase to degrade angiotensin II (Ang II) to angiotensin 1–7 (Ang 1–7) and improves the pathologies of cardiovascular disease and acute respiratory distress syndrome (ARDS)/acute lung injury. B38-CAP is a bacteria-derived ACE2-like carboxypeptidase as potent as human ACE2 and ameliorates hypertension, heart failure and SARS-CoV-2-induced lung injury in mice. Recombinant B38-CAP is prepared with *E*. *coli* protein expression system more efficiently than recombinant soluble human ACE2. Here we show therapeutic effects of B38-CAP on abdominal sepsis- or acid aspiration-induced acute lung injury. ACE2 expression was downregulated in the lungs of mice with cecal ligation puncture (CLP)-induced sepsis or acid-induced lung injury thereby leading to upregulation of Ang II levels. Intraperitoneal injection of B38-CAP significantly decreased Ang II levels while upregulated angiotensin 1–7 levels. B38-CAP improved survival rate of the mice under sepsis. B38-CAP suppressed the pathologies of lung inflammation, improved lung dysfunction and downregulated elevated cytokine mRNA levels in the mice with acute lung injury. Thus, systemic treatment with an ACE2-like enzyme might be a potential therapeutic strategy for the patients with severe sepsis or ARDS.

## Introduction

Acute respiratory distress syndrome (ARDS) or acute lung injury is a life-threatening devastative condition with acute respiratory failure, which is caused by multiple reasons, aspiration pneumonia, sepsis, infection, trauma or major surgery. It has the characteristics of acute onset of diffuse inflammatory lung pathologies, which increase alveolar‐capillary permeability leading to lung edema [1, 2]. ARDS has a high mortality rate (30–40%), with more severe lung injury at the onset being associated with a higher mortality rate (46%) [1, 3]. Despite improvements in the treatment of ARDS, such as fluid control and mechanical ventilation, there are still limitations for therapeutic outcomes [4]. No effective drugs for treating ARDS have been developed so far. Thus, it is necessary to find new therapeutic strategy and drug candidates for treating ARDS/acute lung injury.

The renin–angiotensin system (RAS) maintains cardiovascular homeostasis including blood pressure and fluid balance [5, 6], while it worsens several diseases, including heart failure, myocardial infarction, hypertension, acute lung injury and diabetes [7, 8]. In the RAS, angiotensin-converting enzyme (ACE) is a carboxypeptidase to convert angiotensin I to a vasopressor peptide angiotensin II (Ang II). ACE2 is the enzyme homologous to ACE, while it is a negative regulator of the RAS which degrades Ang II to angiotensin 1–7 (Ang 1–7), thereby counterbalancing ACE activity [9, 10]. In addition to its enzyme activity, ACE2 is a receptor for cell entry of severe acute respiratory syndrome coronavirus 2 (SARS-CoV-2) as well as the 2003 SARS coronavirus [11–13]. Activation of the RAS worsens the pathologies of ARDS/acute lung injury [14], and its mechanisms are suggested to include enhanced adhesion of leukocyte to pulmonary endothelial cells, increased production of inflammatory cytokines through NF-kB activation and increased permeability due to elevated vascular tones and inflammation [14–16]. Infection of SARS-CoV-2 or 2003 SARS coronavirus directly downregulates ACE2 expression in the lungs, which is partly causative of severe lung injuries in SARS-CoV-2 or SARS coronavirus infection [12, 17]. In addition, ACE2 downregulation is also indirectly induced by other causes of lung injury including acid aspiration, sepsis and lethal influenza infection [12, 14, 18]. Genetic deletion of ACE2 augments acute lung injury through Ang II upregulation in mice, whereas treatment with recombinant soluble human ACE2 protein (rshACE2) improves lung injury in animal models [14].

Based on the early studies of lung-protective effects of rshACE2 in acute lung injury, rshACE2 was developed as therapeutics for ARDS patients and tested in the clinic [19]. However, high costs of production of rshACE2 in protein expression system with mammalian cells hampered to obtain conclusive results of the clinical trial for ARDS patients [19]. In search for alternate ACE2-like molecule, which is prepared in *E*. *coli* protein expression system, we have recently identified a novel carboxypeptidase B38-CAP as an ACE2-like enzyme from *Paenibacillus sp*. B38, a new substrain of *Bacillus subtilis* [20]. Recombinant B38-CAP protein catalyzes the conversion of Ang II to Ang 1–7 with the same potency as ACE2 [20]. Treatment with B38-CAP downregulates Ang II levels in mice thereby improving hypertension and heart failure without overt toxicities [20]. In addition, SARS-CoV-2 infection-induced severe lung injury was also significantly improved by B38-CAP through its enzymatic activity [17]. Thus, B38-CAP is anticipated to suppress acute lung injury caused by other diseases.

In this study, to investigate therapeutic effects of B38-CAP in ARDS/acute lung injury, we employed two pre-clinical models of acute lung injury in mice. One is hydrochloric acid (HCl)-aspiration induced acute lung injury, which directly insults lung epithelial cells and causes sterile inflammation, mimicking the clinical situation of inhalation of acidic gastric contents [21, 22]. The second one is the cecal ligation puncture (CLP) model, in which puncture of the cecum causes bacterial peritonitis and induces polymicrobial sepsis thereby leading to increased vascular permeability and severe inflammation in the lungs [23]. We here show that B38-CAP improves sepsis- or acid inhalation-induced severe acute lung injury through downregulation of Ang II levels. Importantly, B38-CAP ameliorated survival rate of the mice with CLP-induced sepsis and ARDS. B38-CAP suppressed pulmonary edema and downregulated high cytokine mRNA levels in the lungs.

## Materials and methods

### Mice

Five weeks-old ICR wild-type male mice were purchased from CLEA Japan, Inc. and maintained at the animal facilities of Akita University Graduate School of Medicine. All animal experiments conformed to the Guide for the Care and Use of Laboratory Animals, Eighth Edition, updated by the US National Research Council Committee in 2011, and approvals of the experiments were granted by the ethics review board of Akita University.

### Recombinant proteins

Recombinant B38-CAP protein was expressed in *E*.*coli*. and purified as previously described [20]. B38-CAP protein was confirmed free of endotoxin contamination (< 0.1 EU μg^−1^ protein) with LAL Endotoxin Assay Kit (Genscript).

### Cecal ligation puncture

To study sepsis-induced acute lung injury, we performed cecal ligation puncture (CLP). Five-week-old male ICR mice were subjected to sepsis by CLP. The peritoneal cavity was cut open under anesthesia. Mice were anesthetized via i.p. injection of ketamine (100 mg kg^−1^) and xylazine (20 mg kg^−1^). Cecum was eviscerated, ligated below the ileocecal valve using 5–0 suture, and punctured through and through with a 21 or 23 gauge needle at three quarters (75% severity) from the end of cecal. The cecum was then squeezed to expel a small amount of fecal material and returned to peritoneal cavity. The abdominal incision was sutured in two layers with 4–0 silk suture. For survival study of septic shock, intraperitoneal injection of B38-CAP (2 mg kg^−1^ per injection) or PBS was started 6 hours after CLP surgery and repeated every 12 hours for 10 days. For B38-CAP treatment in acute lung injury, B38-CAP protein (2 mg kg^−1^) or vehicle was *i*.*p*. injected at 6, 18, 30 and 42 hours after CLP operation. Sham-operated mice underwent the same procedure without ligation and puncture of the cecum. At 48 hours after sham or CLP surgery, animals were euthanized by cervical dislocation.

### Acid-induced acute lung injury

For acid aspiration-induced acute lung injury, five week-old male mice were anesthetized with ketamine (200 mg kg^−1^) and xylazine (10 mg kg^−1^), and were intratracheally instilled with 0.02 M HCl (in 50 μl PBS). For B38-CAP treatment, B38-CAP protein (2 mg kg^−1^) or vehicle was *i*.*p*. injected at 3 and 15 hours after acid instillation. To evaluate lung pathologies, the mice were euthanized with overdose of anesthesia at 24 hours after acid instillation, and blood samples were collected with protease-inhibitor cocktails for Ang II measurements and lungs were excised *en bloc*. After taking macro photography of the lungs, the left lobe of lungs, which usually exhibit more severe injury than right lungs, was cut into three pieces to measure wet to dry weight ratio, RNA expression and protein expression, and the posterior lobe of right lungs was fixed with 4% formalin samples for histological analysis. For measurements of lung function, the mice were anesthetized with ketamine and xylazine at 24 hours after acid aspiration, tracheostomized, mechanically ventilated and lung function measured with Resistance and Compliance system (Buxco). Pulmonary function parameters; elastance, resistance and dynamic or static compliance were obtained under mechanical ventilation with tidal volume (10 ml kg^−1^) and PEEP (2 cm H_2_O) for 15 minutes as previously described [14].

### Histology

Lung tissues were fixed with 4% formalin and embedded in parafin. Five-μm-thick sections were prepared and stained with Hematoxylin & Eosin (H&E). For semi quantitative assessment of lung injury, the high-resolution images (x200 magnification) of the lung sections stained with H&E were taken with microscope (Nikon). Three randomly chosen fields of each section were scored for Lung injury score in a blinded fashion using a previously defined score consisting of alveolar congestion, hemorrhage, neutrophil infiltration, thickness of alveolar wall, and hyaline membrane formation, as follows: 0 = minimal (little) damage, 1 = mild damage, 2 = moderate damage, 3 = severe damage and 4 = maximal damage [24]. The average Lung injury score of three fields of each section were used as the one individual Lung injury score.

### Western blotting

Lung proteins were extracted with TNE lysis buffer (50 mM Tris, 150 mM NaCl, 1 mM EDTA, 1% NP40, protease inhibitor (Complete Mini; Roche) using Microsmash (MS-100R; TOMY), 20 mM NaF, 2 mM Na_3_VO_4_), were sonicated and denatured with LDS sample buffer (Invitrogen) at 70°C. Proteins were electrophoresed on NuPAGE bis-tris precast gels (Invitrogen) and transferred to nitrocellulose membranes (Invitrogen). Membranes were probed with anti-mouse ACE2 antibody [20] and anti-mouse GAPDH antibody (Cell Signaling TECHNOLOGY, 14C10). The blotting bands visualized with ECL reagent (Bio-Rad) using ChemiDoc Touch Imaging System (Bio-Rad). Image Lab software was used to quantify band intensity.

### Measurements of cytokine mRNA expression by qRT-PCR

qRT-PCR analysis was conducted as previously described [17]. Briey, total RNA was extracted using TRIzol reagent (Invitrogen) and cDNA synthesized using the PrimeScript RT reagent kit (RR037; TAKARA). Quantitative real-time PCR was run in 96 well plates using a SYBR Premix ExTaq II (RR820; TAKARA) according to the instructions of the manufacturer. Relative gene expression levels were quantified by using the Thermal Cycler Dice Real Time System II software (TAKARA). Sequences of the forward and reverse primers of the genes studied are shown in S1 Table.

### Measurements of plasma Ang II and Ang 1–7 levels

Plasma Ang II and Ang 1–7 levels were measured with ELISA assay after peptide extraction as previously described [20]. Briefly, blood samples were collected in tubes containing EDTA (25 mM), o-phenanthroline (0.44 mM), pepstatin A (0.12 mM), and p-hydroxymercuribenzoic acid (1 mM), and then centrifuged at 1,200 x g for 10 min. Angiotensin peptides were acidified and extracted with Sep-Pak cartridges (Waters), and Ang II in the peptide extract was quantified using an ELISA kit (Enzo Life Sciences). Plasma Ang 1–7 concentration was also determined by subjecting the peptide extracts to measurements with Ang 1–7 ELISA kit (CUSABIO Biotech).

### Statistical analyses

Data are presented as mean values ± SEM. Statistical significance between two experimental groups was determined using Student’s two-tailed t-test. When a comparison is done for groups, one-way ANOVA with Sidak’s multiple comparisons test were used. P < 0.05 was considered significant.

## Results and discussion

### ACE2 downregulation in mouse lungs under abdominal sepsis or acid-induced acute lung injury

**Fig 1:**
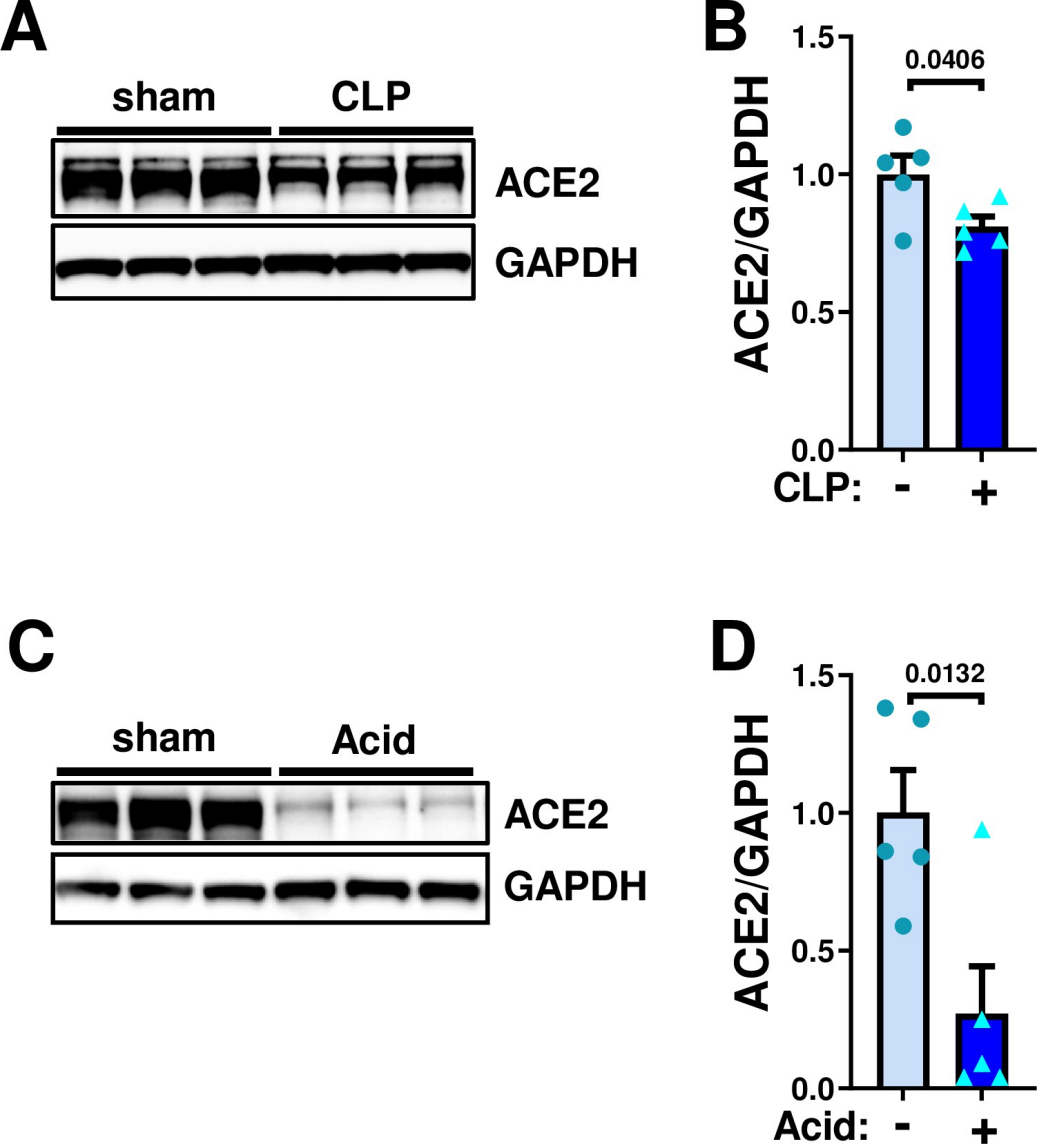
Downregulation of ACE2 expression in mouse lung under sepsis- or acid aspiration-induced lung injury. **A-B**, Protein expression of ACE2 in mouse lungs under sepsis at 48 hours after introducing cecal ligation puncture (CLP)-induced lung injury. Representative Western blot (**A**) and quantification of ACE2 protein abundance (**B**); sham control (n = 5) and CLP surgery (n = 5). **C-D**, Protein expression of ACE2 in mouse lungs at 24 hours after intra-tracheal instillation of acid in mice (0.02M HCl, 50 μl per body). Representative Western blot (**C**) and quantification of ACE2 protein abundance (**D**); sham control (n = 5) and acid inhalation (n = 5). All values are means ± SEM. Two-tailed unpaired t-test.

In our early study, ACE2 expression was downregulated in the lungs under mechanical ventilation with severe lung injury in the very acute phase at 3 hours to overnight after onset of the diseases [14]. To address ACE2 expression at later time points without mechanical ventilation, we first examined expression of ACE2 protein in the lungs of mice with cecal ligation puncture (CLP)-induced septic peritonitis. At 48 hours after CLP surgery, protein expression of ACE2 was significantly downregulated in the lungs compared with sham surgery (Fig 1A and 1B). We next examined ACE2 expression in acid aspiration-induced acute lung injury. At 24 hours after acid aspiration, ACE2 expression in the lungs were significantly decreased (Fig 1C and 1D). Thus, both lung injuries downregulate ACE2 expression in the lungs without mechanical ventilation.

### B38-CAP improves survival rate of the mice under sepsis

**Fig 2:**
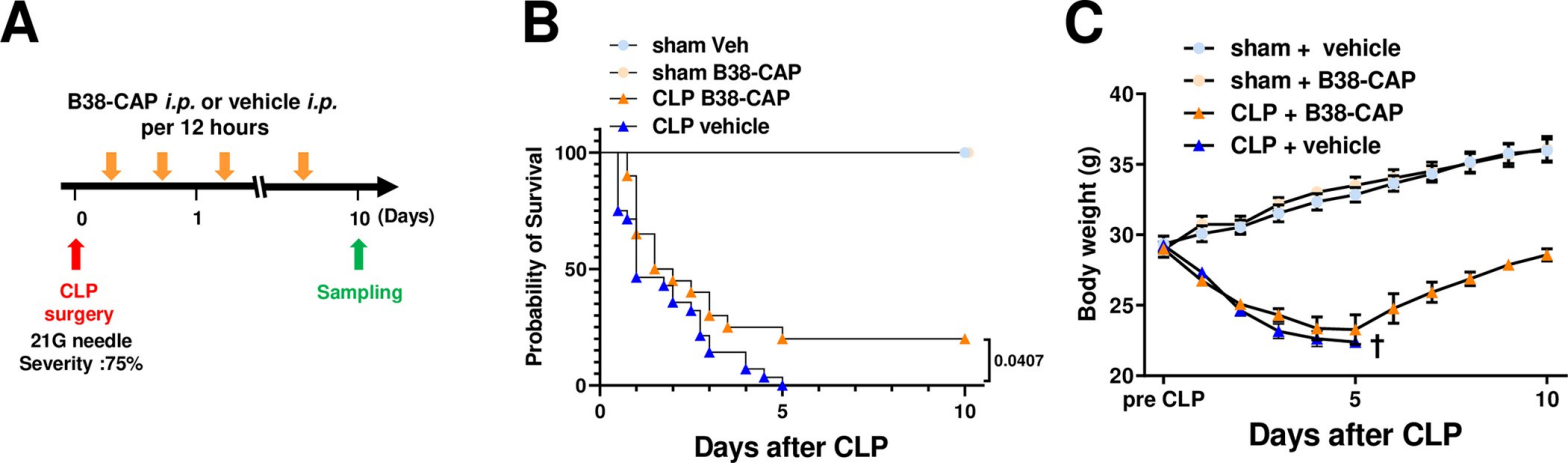
B38-CAP improves mortality of mice with CLP-induced abdominal sepsis. **A**, Experimental protocol of B38-CAP treatment for mice with CLP-induced sepsis. Intraperitoneal injection of B38-CAP (2 mg kg^-1^ pre *i*.*p*.) or vehicle were initiated at 6 hours after CLP surgery and repeated every 12 hours for 10 days. sham + vehicle (n = 6), sham + B38-CAP (n = 6), CLP +　 vehicle (n = 20) and CLP + B38-CAP (n = 28). **B**, Kaplan-Meier survival curves. P < 0.05 (log-rank test) when comparing the CLP + B38-CAP group with the CLP + vehicle group. **C**, Body weight change after CLP induction. All values are means ± SEM.

To address effects of B38-CAP on sepsis, we treated the mice under sepsis with B38-CAP. At 6 hours after CLP surgery, we initiated intraperitoneal (i.p.) injection of B38-CAP (2mg kg^−1^ per injection) or PBS to the mice and treated the mice every 12 hours for 10 days (Fig 2A). This is the effective dose of B38-CAP as an ACE2-like enzyme for treating heart failure and COVID-19-induced lung injury in our previous studies [17, 20]. The administration of B38-CAP prolonged the overall survival time of mice with CLP-induced abdominal sepsis (Fig 2B). Decreased body weight of survived B38-CAP treated mice was recovered (Fig 2C). Thus, B38-CAP is protective against CLP-induced lethal sepsis.

### Suppression of sepsis-induced lung injury by B38-CAP

**Fig 3:**
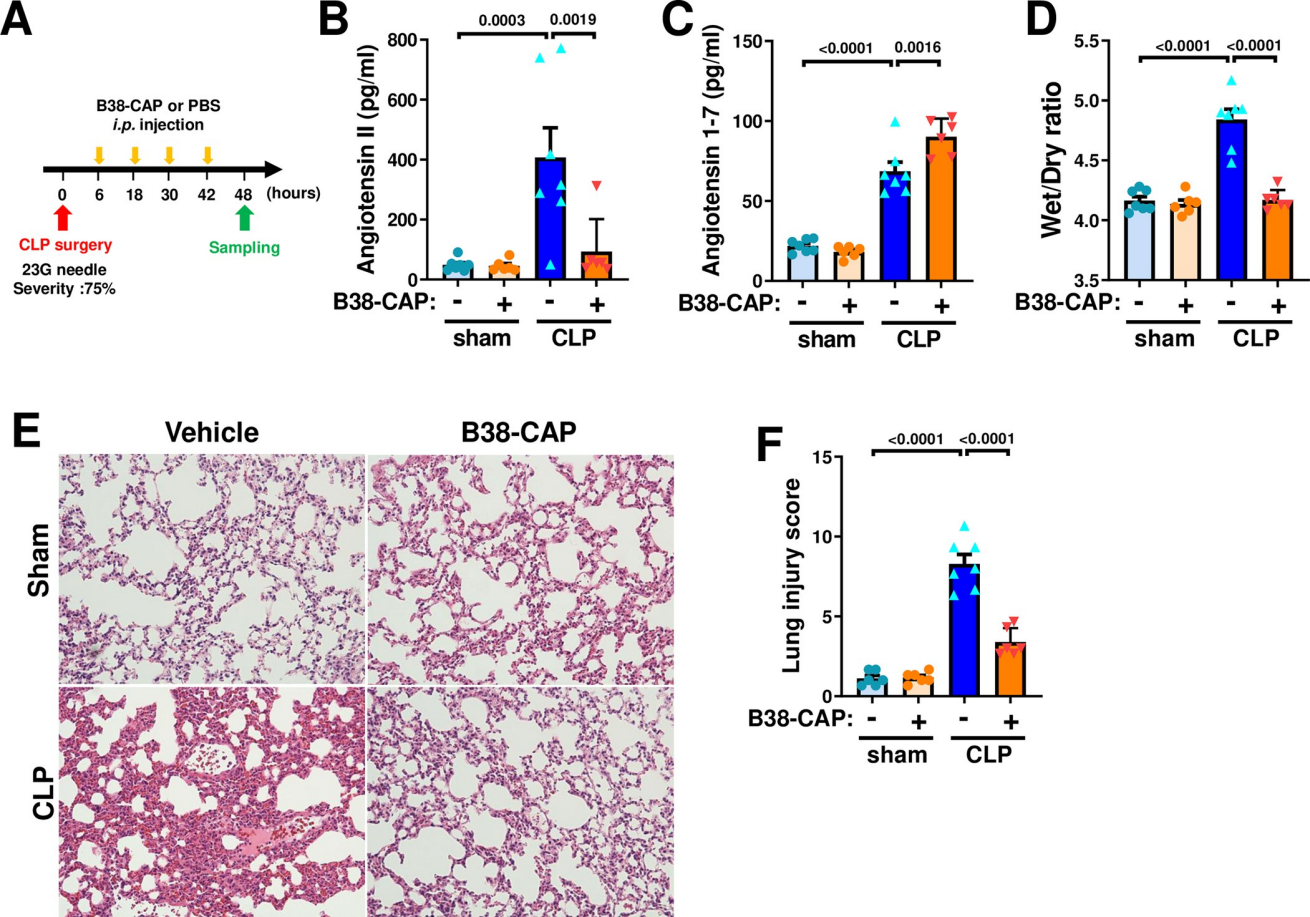
Effects of B38-CAP on lung injury induced by CLP in mice. **A**, Experimental protocol. The mice with CLP were treated with B38-CAP (2 mg kg^−1^ per day) or vehicle, and samples were harvested at 48 hours after CLP surgery. sham + vehicle (n = 7), sham + B38- CAP (n = 6), CLP + vehicle (n = 7) and CLP + B38-CAP (n = 6). **B**, Measurements of plasma Ang II in the mice. **C,** Ang 1–7 levels in the plasma. **D,** Wet to dry weight ratio for lung edema. **E-F**, Lung histopathology. Representative images are shown (**E**). Bars indicate 100 μm. Lung injury score measurements (**F**). All values are means ± SEM. One-way ANOVA with Sidak’s multiple-comparisons test. Numbers above square brackets show significant P-values.

**Fig 4:**
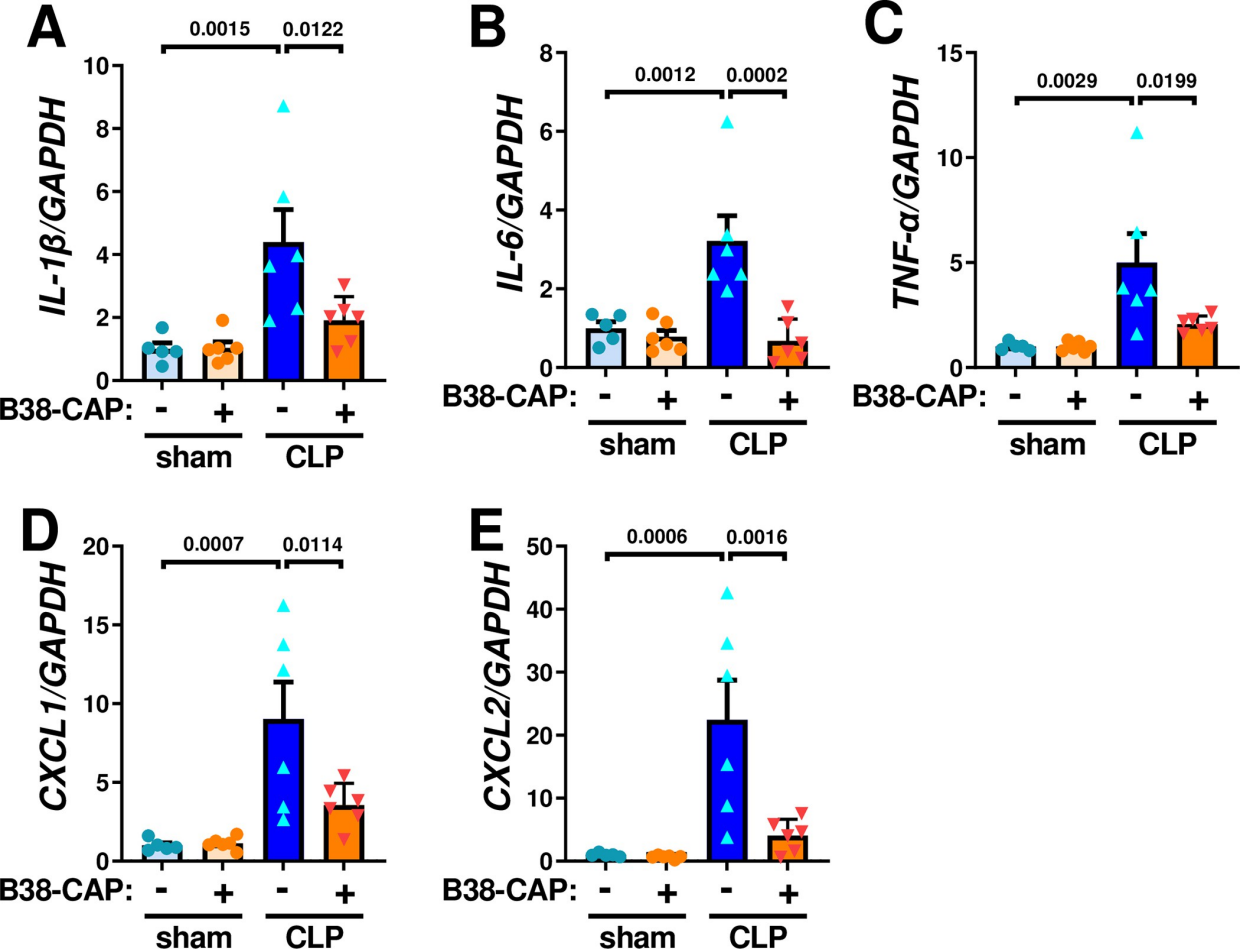
B38-CAP suppresses CLP-induced pulmonary pro-inflammatory cytokines. **A–E**, qRT-PCR analysis for the expression pro-inflammatory cytokine genes in the lungs; mRNA levels of interleukin 1 beta (IL-1β) (**A**), interleukin 6 (IL-6) (**B**), tumor necrosis factor alpha (TNF-α) (**C**), CXCL1 (**D**) and CXCL2 (**E**) in the lungs of mice treated with sham + vehicle (n = 5), sham + B38-CAP (n = 6), CLP + vehicle (n = 6) and CLP + B38-CAP (n = 6). All values are means ± SEM. One-way ANOVA with Sidak’s multiple-comparisons test. Numbers above square brackets show significant P-values.

To examine effects of B38-CAP on sepsis-induced acute lung injury, we harvested lung tissues and plasma from B38-CAP-tretaed mice at 48 hours after CLP surgery (Fig 3A). Elevation of plasma Ang II levels was significantly suppressed by B38-CAP treatment (Fig 3B). Consistently, Ang 1–7 levels in the plasma were significantly upregulated in CLP plus B38-CAP treated mice compared with vehicle treated mice (Fig 3C). At 48 hours after CLP surgery, lung edema as defined by wet weight to dry weight ratio of the lungs (Wet/Dry ratio) was significantly decreased in the B38-CAP-treated mice as compared with vehicle-treated controls (Fig 3D). In addition, B38-CAP treatment suppressed lung inflammation in the mice with CLP, shown as a reduction of lung injury score in the histology which measures the severity of alveolar congestion, hemorrhage, neutrophil infiltration, thickness of alveolar wall, and hyaline membrane formation (Fig 3E and 3F). We further examined mRNA expression of cytokines. While mRNA expression of IL-1β, IL-6, TNF-α, CXCL1 and CXCL2 were upregulated in the lungs of mice with CLP-induced sepsis, B38-CAP treatment significantly downregulated high cytokine mRNA levels (Fig 4A–4E). Thus, B38-CAP suppresses CLP-induced acute lung injury.

### B38-CAP mitigates acid aspiration-induced lung injury

**Fig 5:**
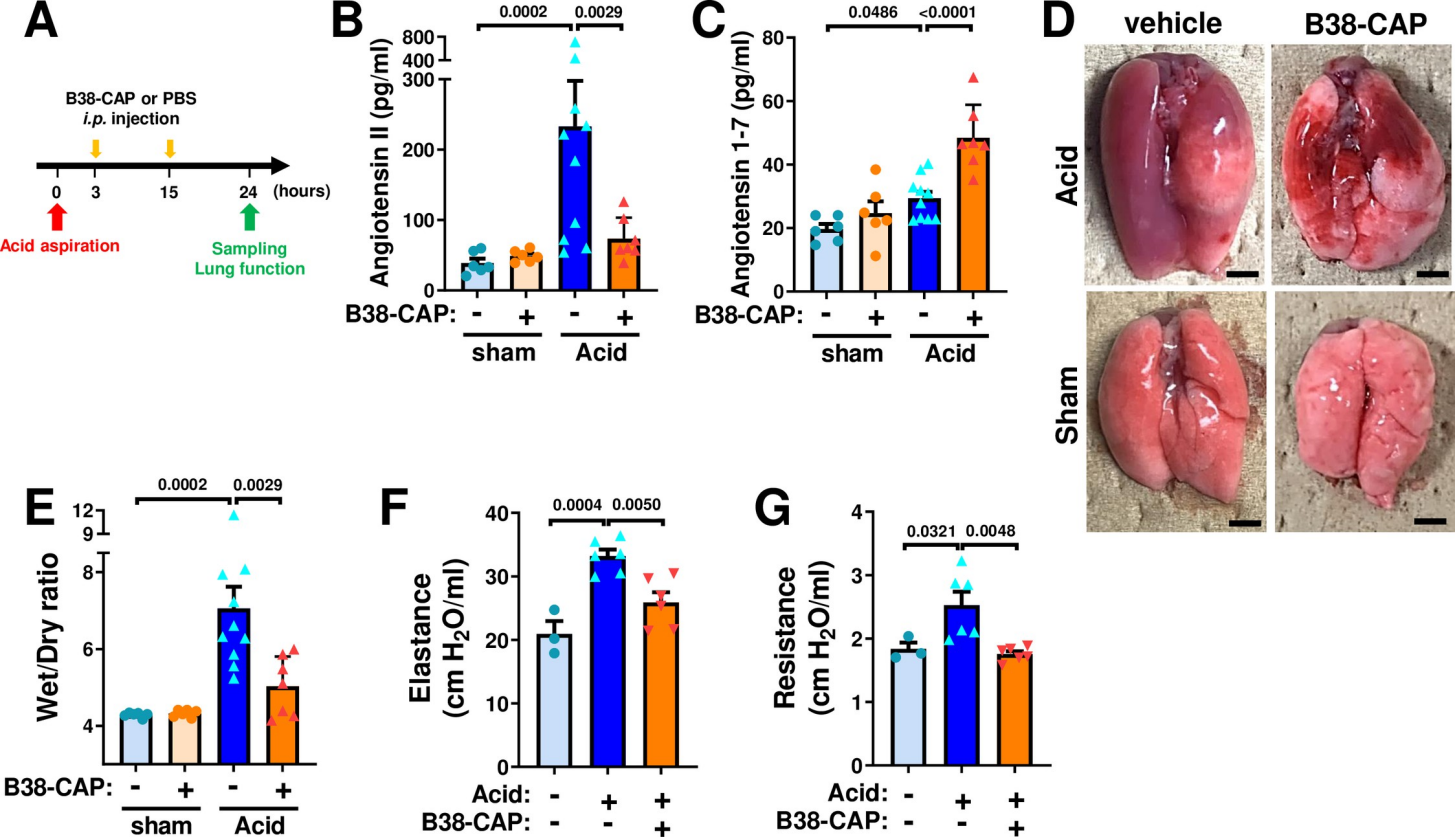
B38-CAP mitigates acid aspiration-induced lung injury. **A**, Experimental protocol. Mice were intra-tracheally instilled with acid (0.02M HCl, 50 μl per body) and were injected with B38-CAP (2 mg/kg *i*.*p*.). **B**, Plasma Ang II measurements. **C**, Ang 1–7 levels in the plasma. **D**, Representative photograph of mouse lungs. Bars indicate 2 mm. **E**, Wet to dry weight ratios of lungs. sham + vehicle (n = 6), sham + B38- CAP (n = 6), Acid + vehicle (n = 10) and Acid + B38-CAP (n = 7) (**B-E**). **F**-**G**, Lung function measurements at 24 hours after acid instillation. Measurements of elastance (**F**) and resistance (**G**) are shown with sham + vehicle (n = 3), Acid + vehicle (n = 6) and Acid + B38-CAP (n = 6). All values are means ± SEM. One-way ANOVA with Sidak’s multiple-comparisons test. Numbers above square brackets show significant P-values.

**Fig 6:**
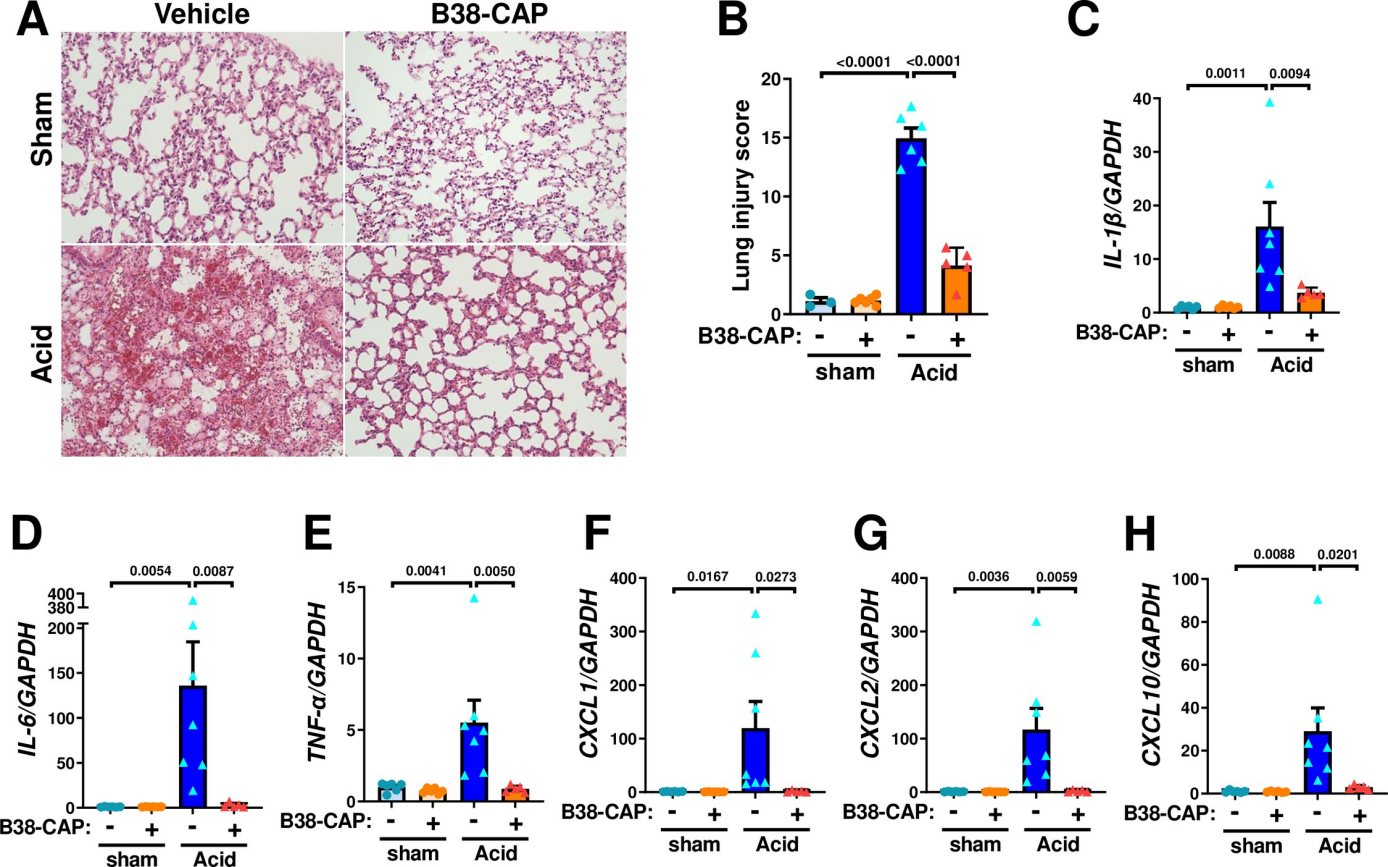
B38-CAP ameliorates acid instillation-induced lung inflammatory and cytokine storm. **A-B**, Lung histopathology. Tissue samples were harvested at 24 hours after acid instillation. Representative images are shown (**A**). Bars indicate 100 μm. Lung injury score measurements (**B**) in the lungs of mice treated with sham + vehicle (n = 3), sham + B38- CAP (n = 6), Acid + vehicle (n = 6), and Acid + B38-CAP (n = 5). **C-H**, qRT-PCR analysis of pro-inflammatory cytokine expression in the lungs of mice; mRNA levels of IL-1β (**C**), IL-6 (**D**), TNF-α (**E**), CXCL1 (**F**), CXCL2 (**G**) and CXCL10 (**H**) normalized with GAPDH. sham + vehicle (n = 6), sham + B38- CAP (n = 6), Acid + vehicle (n = 7), and Acid + B38-CAP (n = 5). All values are means ± SEM. One-way ANOVA with Sidak’s multiple-comparisons test. Numbers above square brackets show significant P-values.

To address the effects of B38-CAP in acid inhalation-induced acute lung injury, mice were instilled acid thorough trachea and treated with intraperitoneal injection of B38-CAP (2mg kg^−1^ per injection) or PBS at 3 hours and 15 hours after acid aspiration (Fig 5A). At 24 hours after acid aspiration, plasma Ang II was increased by acid-induced lung injury, whereas B38-CAP treatment markedly downregulated plasma Ang II levels and increased plasma Ang 1–7 levels (Fig 5B and 5C). The lungs appeared markedly reddish and swollen with hemorrhage in the mice with acid instillation, whereas B38-CAP improved acid-induced lung inflammation and hemorrhage (Fig 5D). Acid aspiration markedly increased wet to dry weight ratio of the lungs, which was significantly decreased by B38-CAP treatment, indicating that B38-CAP suppressed lung edema (Fig 5E). In lung function measurements, acid instillation-induced impairment of lung function as indicated by increase of lung elastance and airway resistance was significantly improved by B38-CAP treatment (Fig 5F and 5G). Histological analysis demonstrated that B38-CAP treatment improved alveolar congestion, hemorrhage and neutrophil infiltration in the lungs of acid induced lung injury (Fig 6A and 6B). Consistently, elevated mRNA levels of cytokines IL-1β, IL-6, TNF-α, CXCL1, CXCL2 and CXCL10 in the lungs of mice with lung injury were markedly downregulated by B38-CAP treatment (Fig 6C–6H). These results indicate that exogenous B38-CAP treatment protects mice from acid aspiration induced pulmonary edema, hemorrhage and inflammation through ACE2-like enzymatic activity.

In this study, we demonstrated that B38-CAP treatment improved mortality of abdominal sepsis in mice. B38-CAP as an ACE2-like enzyme suppresses sepsis- or acid aspiration-induced lung injury associated with decreased Ang II levels and increased Ang 1–7 levels in circulation. B38-CAP markedly downregulated cytokine mRNA levels in the lungs with acute injuries.

B38-CAP is a bacteria-derived protein. Although there are no toxicities of B38-CAP in mice, it might be still anticipated that systemic treatment with B38-CAP may cause unexpected side effects in humans. While beneficial effects of B38-CAP are likely mediated through marked downregulation of Ang II in blood, local down-modulation of Ang II in lung tissues might be also important for its therapeutic effects. In fact, our previous study showed that SARS-CoV-2-induced lung injury did not elevate plasma Ang II levels so high as acid or sepsis-induced lung injury, but that Ang II levels in the lung tissues were markedly upregulated by SARS-CoV-2, which was significantly downregulated by B38-CAP treatment leading to improvement of the lung injury [17]. In addition, inhibition of Ang II signaling in pulmonary epithelial cells has been suggested as lung-protective [25, 26]. Thus, direct delivery of B38-CAP to injured lungs locally, such as inhalation of B38-CAP as aerosols, would be also another option of therapeutic strategy.

In the lungs, acid aspiration-induced lung injury is primarily caused by insults of lung epithelial cells or alveolar compartments, whereas CLP-induced lung injury is caused through endothelial cells or capillary vascular compartment by systemic inflammation. Since therapeutic effects of B38-CAP were observed in these two distinct models, it might be speculated that B38-CAP provides beneficial effects to both alveolar and capillary compartments. It is noteworthy that survival rate of the mice with CLP was significantly improved by B38-CAP treatment. Angiotensin receptor blocker Losartan had been also reported to increase survival rate of the mice with CLP, likely through suppression of NF-kB and MAPK in the lungs [27]. There are few studies addressing which tissues are important for the beneficial effects of Ang II inhibition in sepsis models. Recently, it was reported that genetic deletion of hepatic angiotensinogen, a precursor of Ang II, attenuated CLP-induced myocardial dysfunction and increased survival rate [28]. Comprehensive analyses for RAS activation in lungs as well as other organs, including liver, heart and kidney, would be necessary. On the other hand, because B38-CAP was administered by intraperitoneal injection, it might be speculated that B38-CAP directly affect the pathologies of polymicrobial peritonitis. For instance, given that hyper-activation of immune system in the peritonitis worsen the pathologies of sepsis in this model, B38-CAP might be beneficial in down-modulating unwanted inflammation without affecting bacterial elimination thereby improving survival. Further detailed analyses for unknown roles of ACE2 and B38-CAP in peritonitis/sepsis would be necessary.

Clinical studies have suggested that ARB or ACE inhibitor provide better prognosis to the COVID-19 patients as well as ARDS patients in general [29–31]. As discussed in our recent paper, the viewpoint that the metabolites generated by and other substrates for ACE2 might also play roles in ameliorating lung injury should be noted [17]. The enzymatic activity of both B38-CAP and ACE2 does not only degrade Ang II but also generates Ang 1–7. Binding of Ang 1–7 to its cognate receptor Mas is suggested to protect from acute lung injury [32]. In addition, both ACE2 and B38-CAP target other peptide for its substrates. For instance, des-Arg^9^-bradykinin is a substrate for ACE2 and an agonist of B1 bradykinin receptor, and activation of the B1 receptor by attenuation of ACE2 activity promotes vascular permeability and inflammation in LPS-induced lung injury [33]. Thus, the beneficial effect of ACE2 enzymatic activity in lung injury might contain the degradation of des-Arg^9^-bradykinin in addition to Ang II [17]. On the other hand, another ACE2 substrate Apelin conversely ameliorates lung injury [34], whereas ACE2 inactivates Apelin [35]. Furthermore, Apelin increases expression of ACE2 in failing hearts [36], if this is the case for lung injury, making it more complicated. There seems a complicated network of ACE2 and its substrate peptides. Further analyses are warranted to elucidate the mechanisms for therapeutic effects of ACE2 and B38-CAP in acute lung injury.

## Conclusion

While soluble ACE2 functional as both lung-protecting enzyme and decoy for SARS-CoV-2 might be good for COVID-19 treatment, the ACE2 enzymatic activity of B38-CAP is likely sufficient for treating ARDS induced by other causes. Enhancement of ACE2 enzymatic activity by B38-CAP is a potential therapeutic strategy to alleviate the symptoms of ARDS in sepsis or aspiration pneumonia.

## Supporting information

S1 Fig Uncropped membranes of Western blot. **A**, Uncropped images of the blot in Fig 1A. **B**, Uncropped images of the blot in Fig 1C.
(TIF)

S1 Table qRT-PCR primers for cytokine mRNA measurements. (DOCX)
