# Supplementary figures and images for: ACE2-like enzyme B38-CAP suppresses abdominal sepsis and severe acute lung injury
Source: PLoS One. 2022 Jul 22;17(7):e0270920. doi: 10.1371/journal.pone.0270920 (PMC9307200; doi:10.1371/journal.pone.0270920)

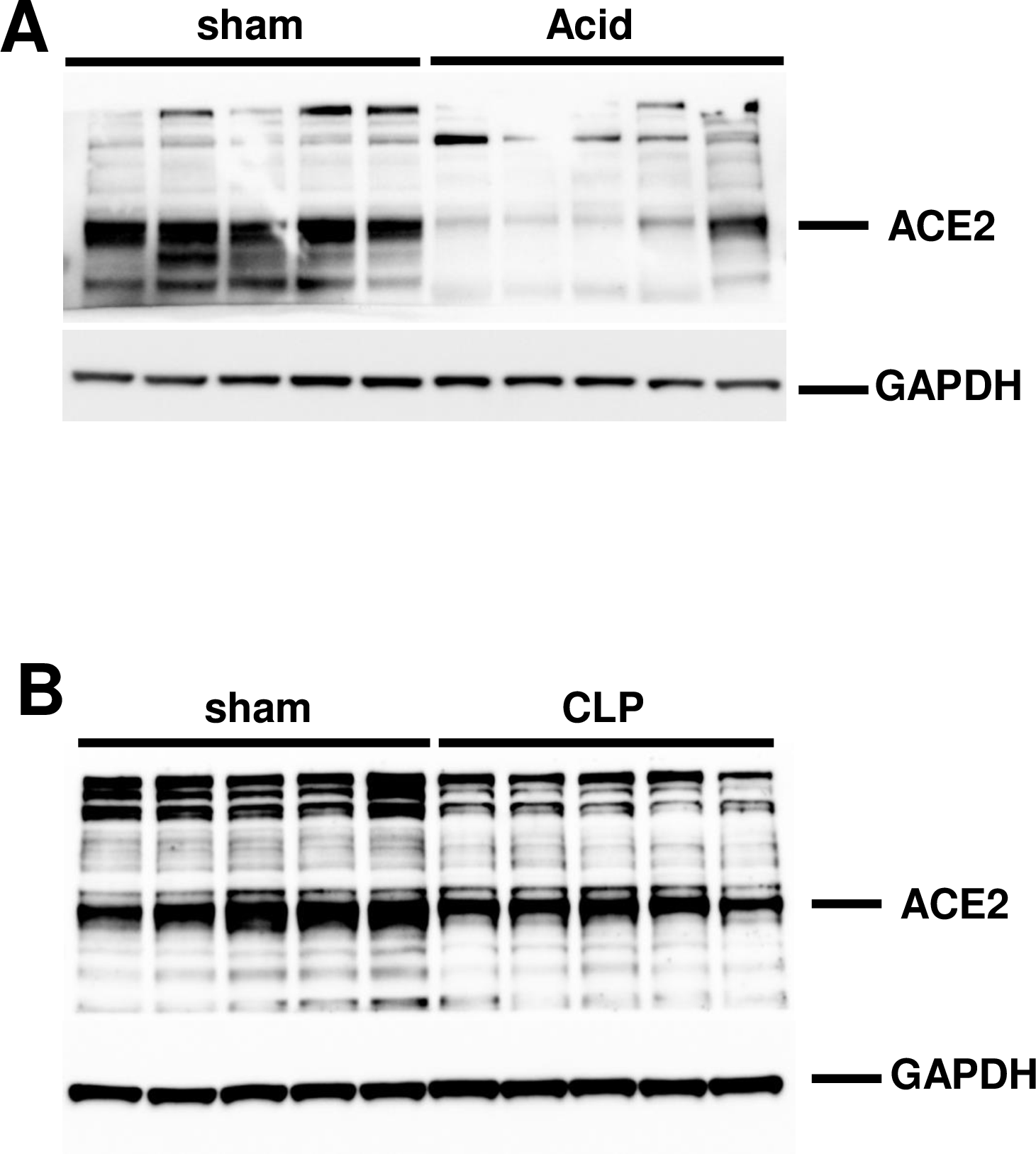

Supplement: S1 Fig — A, Uncropped images of the blot in Fig 1A. B, Uncropped images of the blot in Fig 1C. (TIF) [file pone.0270920.s001.tif]
