# Supplementary material for: ACE2-like enzyme B38-CAP suppresses abdominal sepsis and severe acute lung injury
Source: PLoS One. 2022 Jul 22;17(7):e0270920. doi: 10.1371/journal.pone.0270920 (PMC9307200; doi:10.1371/journal.pone.0270920)
Supplement: S1 Table — (DOCX) [file pone.0270920.s002.docx]

| genes | 5'-Sense-3' | 5'-Antisense-3' |
| --- | --- | --- |
| *IL-1β* | GGACAATGACTATGTGTTGTTAGAA | AGGCAAATTTCCCAATTGTATCCAG |
| *IL-6* | CAACGATGATGCACTTGCAGA | CTCCAGGTAGCTATGGTACTCCAGA |
| *TNF-α* | GCCTCTTCTCATTCCTGCTTG | CTGATGAGAGGGAGGCCATT |
| *CXCL1* | GCTTGAAGGTGTTGCCCTCAG | AAGCCTCGCGACCATTCTTG |
| *CXCL2* | GCGCTGTCAATGCCTGAAGA | TTTGACCGCCCTTGAGAGTG |
| *CXCL10* | GCCGTCATTTTCTGCCTCAT | GCTTCCCTATGGCCCTCATT |
| *GAPDH* | CTGCACCACCAACTGCTTAG | GTCTTCTGGGTGGCAGTGAT |

**S1 Table. qRT-PCR primers for cytokine mRNA measurements**
